# Identify biological Alzheimer’s disease using a novel nucleic acid–linked protein immunoassay

**DOI:** 10.1093/braincomms/fcaf004

**Published:** 2025-01-07

**Authors:** Yi-Ting Wang, Nicholas J Ashton, Joseph Therriault, Andréa L Benedet, Arthur C Macedo, Ilaria Pola, Etienne Aumont, Guglielmo Di Molfetta, Jaime Fernandez-Arias, Kubra Tan, Nesrine Rahmouni, Stijn Johannes G Servaes, Richard Isaacson, Tevy Chan, Seyyed Ali Hosseini, Cécile Tissot, Sulantha Mathotaarachchi, Jenna Stevenson, Firoza Z Lussier, Tharick A Pascoal, Serge Gauthier, Kaj Blennow, Henrik Zetterberg, Pedro Rosa-Neto

**Affiliations:** Translational Neuroimaging Laboratory, McGill University Research Centre for Studies in Aging, Montreal, QC, Canada H4H 1R2; Montreal Neurological Institute, Montreal, QC, Canada H3A 2B4; Department of Neurology and Neurosurgery, Faculty of Medicine, McGill University, Montreal, QC, Canada H3A 0G4; Department of Psychiatry and Neurochemistry, Institute of Neuroscience and Physiology, The Sahlgrenska Academy, University of Gothenburg, 431 39 Mölndal, Sweden; Centre for Age-Related Medicine, Stavanger University Hospital, 4011 Stavanger, Norway; Maurice Wohl Clinical Neuroscience Institute, King’s College London, London SE5 9RX, UK; NIHR Maudsley Biomedical Research Centre for Mental Health and Biomedical Research Unit for Dementia at South London and Maudsley NHS Foundation, London SE5 8AZ, UK; Translational Neuroimaging Laboratory, McGill University Research Centre for Studies in Aging, Montreal, QC, Canada H4H 1R2; Montreal Neurological Institute, Montreal, QC, Canada H3A 2B4; Department of Psychiatry and Neurochemistry, Institute of Neuroscience and Physiology, The Sahlgrenska Academy, University of Gothenburg, 431 39 Mölndal, Sweden; Translational Neuroimaging Laboratory, McGill University Research Centre for Studies in Aging, Montreal, QC, Canada H4H 1R2; Montreal Neurological Institute, Montreal, QC, Canada H3A 2B4; Department of Neurology and Neurosurgery, Faculty of Medicine, McGill University, Montreal, QC, Canada H3A 0G4; Department of Psychiatry and Neurochemistry, Institute of Neuroscience and Physiology, The Sahlgrenska Academy, University of Gothenburg, 431 39 Mölndal, Sweden; Translational Neuroimaging Laboratory, McGill University Research Centre for Studies in Aging, Montreal, QC, Canada H4H 1R2; Montreal Neurological Institute, Montreal, QC, Canada H3A 2B4; Department of Neurology and Neurosurgery, Faculty of Medicine, McGill University, Montreal, QC, Canada H3A 0G4; Department of Psychiatry and Neurochemistry, Institute of Neuroscience and Physiology, The Sahlgrenska Academy, University of Gothenburg, 431 39 Mölndal, Sweden; Translational Neuroimaging Laboratory, McGill University Research Centre for Studies in Aging, Montreal, QC, Canada H4H 1R2; Montreal Neurological Institute, Montreal, QC, Canada H3A 2B4; Department of Neurology and Neurosurgery, Faculty of Medicine, McGill University, Montreal, QC, Canada H3A 0G4; Department of Psychiatry and Neurochemistry, Institute of Neuroscience and Physiology, The Sahlgrenska Academy, University of Gothenburg, 431 39 Mölndal, Sweden; Translational Neuroimaging Laboratory, McGill University Research Centre for Studies in Aging, Montreal, QC, Canada H4H 1R2; Montreal Neurological Institute, Montreal, QC, Canada H3A 2B4; Department of Neurology and Neurosurgery, Faculty of Medicine, McGill University, Montreal, QC, Canada H3A 0G4; Translational Neuroimaging Laboratory, McGill University Research Centre for Studies in Aging, Montreal, QC, Canada H4H 1R2; Department of Neurology, Weill Cornell Medicine and New York-Presbyterian, New York, NY 10065, USA; Department of Neurology, Florida Atlantic University, Charles E. Schmidt College of Medicine, Boca Raton, FL 33431, USA; Translational Neuroimaging Laboratory, McGill University Research Centre for Studies in Aging, Montreal, QC, Canada H4H 1R2; Montreal Neurological Institute, Montreal, QC, Canada H3A 2B4; Translational Neuroimaging Laboratory, McGill University Research Centre for Studies in Aging, Montreal, QC, Canada H4H 1R2; Montreal Neurological Institute, Montreal, QC, Canada H3A 2B4; Department of Neurology and Neurosurgery, Faculty of Medicine, McGill University, Montreal, QC, Canada H3A 0G4; Lawrence Berkeley National Laboratory, Berkeley, CA 94720, USA; Translational Neuroimaging Laboratory, McGill University Research Centre for Studies in Aging, Montreal, QC, Canada H4H 1R2; Translational Neuroimaging Laboratory, McGill University Research Centre for Studies in Aging, Montreal, QC, Canada H4H 1R2; Department of Neurology and Psychiatry, University of Pittsburgh School of Medicine, Pittsburgh, PA 15213, USA; Department of Neurology and Psychiatry, University of Pittsburgh School of Medicine, Pittsburgh, PA 15213, USA; Translational Neuroimaging Laboratory, McGill University Research Centre for Studies in Aging, Montreal, QC, Canada H4H 1R2; Department of Neurology and Neurosurgery, Faculty of Medicine, McGill University, Montreal, QC, Canada H3A 0G4; Department of Psychiatry and Neurochemistry, Institute of Neuroscience and Physiology, The Sahlgrenska Academy, University of Gothenburg, 431 39 Mölndal, Sweden; Clinical Neurochemistry Laboratory, Sahlgrenska University Hospital, 413 45 Mölndal, Sweden; Paris Brain Institute, ICM, Pitié-Salpêtrière Hospital, Sorbonne University, 75013 Paris, France; Neurodegenerative Disorder Research Center, Division of Life Sciences and Medicine and Department of Neurology, Institute on Aging and Brain Disorders, University of Science and Technology of China and First Affiliated Hospital of USTC, Hefei 101127, P. R.China; Department of Psychiatry and Neurochemistry, Institute of Neuroscience and Physiology, The Sahlgrenska Academy, University of Gothenburg, 431 39 Mölndal, Sweden; Clinical Neurochemistry Laboratory, Sahlgrenska University Hospital, 413 45 Mölndal, Sweden; Department of Neurodegenerative Disease, UCL Institute of Neurology, London WC1N 3BG, UK; UK Dementia Research Institute at UCL, London W1CE 6BT, UK; Hong Kong Center for Neurodegenerative Diseases, Hong Kong, China; Wisconsin Alzheimer’s Disease Research Center, University of Wisconsin School of Medicine and Public Health, University of Wisconsin-Madison, Madison, WI 53792, USA; Translational Neuroimaging Laboratory, McGill University Research Centre for Studies in Aging, Montreal, QC, Canada H4H 1R2; Montreal Neurological Institute, Montreal, QC, Canada H3A 2B4; Department of Neurology and Neurosurgery, Faculty of Medicine, McGill University, Montreal, QC, Canada H3A 0G4

**Keywords:** Alzheimer’s disease, blood-based biomarker, PET, head-to-head comparison

## Abstract

Blood-based biomarkers have been revolutionizing the detection, diagnosis and screening of Alzheimer’s disease. Specifically, phosphorylated-tau variants (p-tau_181_, p-tau_217_ and p-tau_231_) are promising biomarkers for identifying Alzheimer’s disease pathology. Antibody-based assays such as single molecule arrays immunoassays are powerful tools to investigate pathological changes indicated by blood-based biomarkers and have been studied extensively in the Alzheimer’s disease research field. A novel proteomic technology—NUcleic acid Linked Immuno-Sandwich Assay (NULISA)—was developed to improve the sensitivity of traditional proximity ligation assays and offer a comprehensive outlook for 120 protein biomarkers in neurodegenerative diseases. Due to the relative novelty of the NULISA technology in quantifying Alzheimer’s disease biomarkers, validation through comparisons with more established methods is required. The main objective of the current study was to determine the capability of p-tau variants quantified using NULISA for identifying abnormal amyloid-β and tau pathology. We assessed 397 participants [mean (standard deviation) age, 64.8 (15.7) years; 244 females (61.5%) and 153 males (38.5%)] from the Translational Biomarkers in Aging and Dementia (TRIAD) cohort where participants had plasma measurements of p-tau_181_, p-tau_217_ and p-tau_231_ from NULISA and single molecule arrays immunoassays. Participants also underwent neuroimaging assessments, including structural MRI, amyloid-PET and tau-PET. Our findings suggest an excellent agreement between plasma p-tau variants quantified using NULISA and single molecule arrays immunoassays. Plasma p-tau_217_ measured with NULISA shows excellent discriminative accuracy for abnormal amyloid-PET (area under the receiver operating characteristic curve = 0.918, 95% confidence interval = 0.883 to 0.953, *P* < 0.0001) and tau-PET (area under the receiver operating characteristic curve = 0.939; 95% confidence interval = 0.909 to 0.969, *P* < 0.0001). It also presents the capability for differentiating tau-PET staging. Validation of the NULISA-measured plasma biomarkers adds to the current analytical methods for Alzheimer’s disease diagnosis, screening and staging and could potentially expedite the development of a blood-based biomarker panel.

## Teaser

Wang *et al.* reported an excellent agreement between the novel NUcleic acid Linked Immuno-Sandwich Assay (NULISA) and single molecule array immunoassays. The multiplexed NULISA assay demonstrates great diagnostic accuracy in identifying biological Alzheimer’s disease, which will add to the current analytical methods for leveraging blood-based biomarkers in diagnosis, screening and staging.

## Graphical Abstract

**Graphical Abstract:**
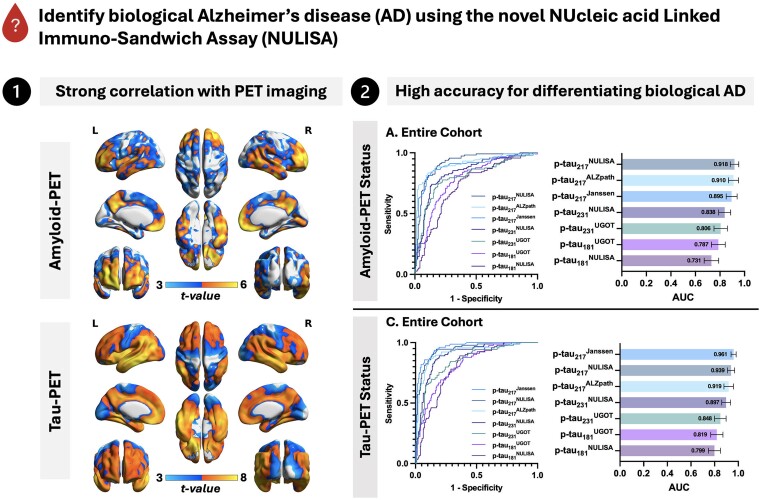

## Introduction

Amyloid-β (Aβ) and tau pathology are the defining pathological features of Alzheimer’s disease.^1^*In vivo* detection of these processes can be done using PET^2^ and quantification of Aβ and phosphorylated-tau (p-tau) proteins in the CSF.^3,4^ Aβ and tau biomarkers are crucial for differential diagnosis,^5,6^ Alzheimer’s disease biological definitions^7,8^ and selection of individuals for clinical trials and disease-modifying therapies.^9,10^ Although PET and CSF biomarkers for Aβ and tau offer high accuracy, their cost and limited availability pose challenges for clinical diagnostic practice and screening in clinical trials. In contrast, the accessibility and cost-effectiveness of blood-based biomarkers make them appealing for first-line clinical use and for facilitating clinical trial recruitment and monitoring.^9,11^

Antibody-based single molecule array (Simoa) immunoassays are powerful tools to investigate pathological changes indicated by blood-based biomarkers and have been used and studied extensively in Alzheimer’s disease research.^12,13^ A novel proteomic technology—NUcleic acid Linked Immuno-Sandwich Assay (NULISA™)^13^—has been developed to offer a broad and in-depth proteomic analysis. By suppressing assay background via a dual capture and release mechanism built into oligonucleotide-conjugated antibodies, NULISA improves the sensitivity of traditional proximity ligation assays.^14^ Additionally, the fully automated multiplexed quantification, which supports the analysis of ∼120 proteins of potential relevance to CNS disease, not only renders it an efficient and cost-effective choice but also offers a more comprehensive outlook for protein biomarker discovery and validation studies in neurodegenerative dementias. Due to the relative novelty of the NULISA technology in quantifying Alzheimer’s disease plasma biomarkers, validation through comparisons with more established methods is required.

The overarching goal of this present study was to validate the diagnostic accuracy of NULISA-quantified plasma p-tau biomarkers for the detection of Alzheimer’s disease Aβ and tau pathology via head-to-head comparisons with Simoa immunoassay-based methods.

## Methods

### Participants

We assessed 397 participants from the Translational Biomarkers of Aging and Dementia (TRIAD) cohort: 206 cognitively unimpaired (CU) older adults, 85 individuals on the Alzheimer’s disease clinical spectrum [42 mild cognitive impairment (MCI) due to Alzheimer’s disease and 43 typical Alzheimer’s disease], 21 atypical Alzheimer’s disease and 58 participants with other neurodegenerative diseases (OND). We also recruited 27 young subjects (age <26 years old). All participants had plasma measurements of p-tau_181_, p-tau_217_ and p-tau_231_ from both NULISA and Simoa immunoassays. The majority of participants (*n* = 337) underwent neuroimaging assessments including MRI and PET scans within 9-month intervals from the blood sample collection. CU individuals had no objective cognitive impairment and a Clinical Dementia Rating (CDR) score of 0. Individuals with MCI had objective cognitive impairment and a CDR score of 0.5. Individuals with dementia due to Alzheimer’s disease (CDR score between 1 and 2) met the National Institute on Aging and Alzheimer’s Association criteria for probable Alzheimer’s disease as determined by a physician. We excluded participants with inadequately treated systemic conditions, active substance abuse, recent head trauma, major surgery or presenting with MRI/PET safety contraindications. The study was approved by the Montreal Neurological Institute PET Working Committee and the Douglas Mental Health University Institute Research Ethics Board. Written informed consent was obtained from all participants. The present study followed the Strengthening the Reporting of Observational Studies in Epidemiology reporting guidelines.

### Brain imaging methodology

Detailed PET imaging acquisition and processing pipelines are described in Supplementary Method 1. Neocortical [^18^F]AZD4694 standardized uptake value ratio (SUVR) was estimated for each participant by averaging the SUVR from the precuneus, pre-frontal, orbitofrontal, parietal, temporal, anterior and posterior cingulate cortices. Tau-PET Braak stage segmentation was previously described.^15,16^ [^18^F]MK6240 meta region of interest (ROI) SUVRs were generated by averaging the SUVR from the entorhinal, amygdala, para-hippocampal, fusiform, inferior temporal and medial temporal regions. Aβ and tau positivity were assigned based on [^18^F]AZD4694 neocortical SUVR (cut-off = 1.55)^17^ and [^18^F]MK6240 meta-ROI SUVR (cut-off = 1.187).^18^

### Plasma p-tau biomarkers

#### p-tau Simoa immunoassays

The plasma collection protocol in the TRIAD cohort followed the procedures previously described.^18^ p-tau biomarkers quantification was performed at the University of Gothenburg (Gothenburg, Sweden). p-tau_181_^UGOT^ and p-tau_231_^UGOT^ were measured using in-house Simoa immunoassays developed at the University of Gothenburg.^19,20^ Plasma p-tau_217_ was measured using Simoa immunoassay developed by Janssen Research and Development (p-tau_217_^Janssen^)^21,22^ and ALZpath (p-tau_217_^ALZpath^).^13^ Details of these p-tau Simoa immunoassays are described in Supplementary Method 2.

#### NULISAseq assay

Plasma samples were analysed using the novel NULISAseq CNS Panel targeting 120 proteins associated with a broad spectrum of neurodegenerative disorders. Next-generation sequencing data were processed using the NULISAseq algorithm (Alamar Biosciences), and intra-plate and inter-plate normalization were performed as described previously.^14^ To facilitate statistical analyses, interpolate control–normalized counts were log_2_ transformed and referred to as NULISA Protein Quantification (NPQ) units. NPQ units are the measurements used for analyses in this study. Information on the NULISAseq assay, data processing and normalization are described in Supplementary Method 3.

### Neuroimaging voxel-based analysis

Young CU subjects were excluded from all the imaging and statistical analyses as they were only used to establish the thresholds to assign the tau-PET status (described in Supplementary Method 4). Voxel-based analyses were performed in MATLAB using the VoxelStats toolbox (https://github.com/sulantha2006/VoxelStats).^23^ The multivariate linear regression models outlined below were conducted in the subjects with neuroimaging assessments (*n* = 310), as well as separately for male and female individuals. The goal was to elucidate the relationship between plasma p-tau biomarker concentrations and PET signals in the brain, while also taking into consideration the potential effects stemming from biological sex.

In every brain voxel, the model test for the relationship between amyloid-PET and plasma biomarkers was of the form:


$$\begin{array}{rl} \mathrm{Amyloid}\text{-}\mathrm{PET}= & \;\beta 0+\beta 1(\mathrm{plasma}\;p\text{-}\mathrm{tau}\;\mathrm{biomarkers})\\ & +\mathrm{covariates}+\varepsilon \end{array}$$

The model test for the relationship between tau-PET and plasma biomarkers was of the form:


Tau-PET=β0+β1(plasmap-taubiomarkers)+covariates+ε

Age and *APOEε4* carriage status were used as covariates in the models. To account for the effects of participants’ biological stage of Alzheimer’s disease, amyloid-PET status (A− or A+) and pathological status (A−T−, A+T− or A+T+) were included as a covariate when assessing the relationship between plasma p-tau biomarkers with amyloid-PET and tau-PET respectively. *t*-statistical parametric maps were corrected for multiple comparisons using a false discovery rate (FDR) threshold of *P* < 0.001. BrainNet Viewer was used to visualize the results from the neuroimaging voxel-based analyses.^24^

### Statistical analysis

Statistical analyses were performed in Python 3.9.12 and GraphPad Prism v9. Group comparisons on continuous dependent demographic and clinical variables were conducted using one-way ANOVA with Tukey’s Honest Significant Difference test to verify group differences. Comparisons on categorical variables were performed using χ^2^ tests. We employed simple linear regression analyses to evaluate the relationship between plasma p-tau biomarkers measured using NULISA and Simoa immunoassays. Additionally, Bland–Altman analyses were performed to assess the agreement between p-tau measurements from NULISA and Simoa immunoassays. To access the diagnostic accuracies of plasma biomarkers, receiver operating characteristic (ROC) curve analysis was conducted, with PET imaging biomarkers serving as the reference standard instead of clinical diagnosis in line with the biological definition of Alzheimer’s disease. The area under the ROC curve (AUC) values were calculated for all p-tau biomarkers (p-tau_181_, p-tau_217_ and p-tau_231_). Finally, multiple logistic regression was performed to investigate the capability of plasma p-tau_217_ biomarkers for predicting an abnormal amyloid-PET and a tau-PET proxy of moderate/high severity (Supplementary Method 4).

## Results

### Participants

The demographic and clinical characteristics of participants involved in this study are reported in Table 1. No significant group differences were found in age, sex or education levels. Individuals within the Alzheimer’s disease spectrum and subjects with atypical Alzheimer’s disease presented a significantly lower score in the mini-mental state examination (MMSE), a significantly higher percentage of *APOEε4* carriers and a substantial number of positive amyloid-PET and tau-PET status. The prevalences of individuals with positive amyloid-PET and positive tau-PET are 41.05% and 31.08%, respectively.

**Table 1:** Demographics and clinical characteristics of participants

	Young	CU	Alzheimer’s disease spectrum	Atypical Alzheimer’s disease	OND
No.	27	206	85	21	58
Sex (female, %)	70.4	66.0	56.5	47.6	53.4
Age, mean (SD), years	22.3 (1.6)	68.3 (10.9)	70.0 (7.8)	66.1 (9.3)	67.9 (9.3)
Education, mean (SD), years	16.3 (1.4)	15.5 (3.5)	15.4 (3.4)	14.1 (4.2)	14.6 (4.3)
MMSE score, mean (SD)	29.9 (0.3)	29.3 (0.9)	25.3 (4.8)	20.6 (5.5)	26.9 (5.1)
APOEε4 carrier (%)	33.3	28.4	61.5	52.6	18
Plasma p-tau biomarker (log_2_ transformed)					
p-tau_181_^NULISA^	11.85 (0.44)	11.99 (0.68)	12.54 (0.58)	12.69 (0.71)	12.11 (0.92)
p-tau_181_^UGOT^	2.35 (0.98)	2.89 (0.83)	3.55 (0.63)	3.86 (0.78)	2.94 (0.85)
p-tau_217_^NULISA^	11.01 (0.36)	11.42 (0.81)	12.77 (0.70)	13.01 (0.80)	11.40 (1.00)
p-tau_217_^Janssen^	−4.75 (0.52)	−4.07 (0.86)	−2.43 (0.91)	−1.93 (1.02)	−4.02 (0.94)
p-tau_217_^ALZpath^	−2.70 (0.52)	−1.73 (1.12)	0.06 (0.88)	0.14 (1.13)	−1.73 (1.25)
p-tau_231_^NULISA^	11.47 (0.46)	11.47 (0.67)	12.33 (0.61)	12.48 (0.67)	11.50 (0.95)
p-tau_231_^UGOT^	3.46 (0.61)	3.76 (0.59)	4.44 (0.58)	4.64 (0.60)	3.84 (0.79)
PET imaging biomarker					
Amyloid-PET neocortical SUVR	1.17 (0.07)	1.44 (0.36)	2.38 (0.41)	2.30 (0.67)	1.24 (0.11)
Amyloid-PET status (positive, %)	0	22.1	98.6	80	0
Tau-PET meta-ROI SUVR	0.91 (0.09)	0.99 (0.21)	1.95 (0.91)	2.59 (1.05)	1.03 (0.54)
Tau-PET status (positive, %)	0	10.1	79.2	88.9	4.3

No significant differences were found in age, sex or education levels among CU, Alzheimer’s disease spectrum, atypical Alzheimer’s disease and OND groups. Individuals within the Alzheimer’s disease spectrum and subjects with atypical Alzheimer’s disease presented a significantly higher percentage of *APOEε4* carriers and a substantial number of positive amyloid-PET and tau-PET status.

### Relationships between p-tau biomarkers quantified using NULISA and Simoa immunoassays

Scatterplots representing log_2_-transformed concentrations of plasma p-tau biomarkers from NULISA and Simoa immunoassays are shown in Supplementary Fig. 1 (top panels). P-tau_217_ measured using different immunoassays presented moderate-strong linear relationships (p-tau_217_^NULISA versus Janssen^: *R*^2^ = 0.73, *P* < 0.0001; p-tau_217_^NULISA versus ALZpath^: *R*^2^ = 0.71, *P* < 0.0001). This relationship appeared weaker for p-tau_181_ and p-tau_231_ (p-tau_181_^NULISA versus UGOT^: *R*^2^ = 0.48; p-tau_231_^NULISA versus UGOT^: *R*^2^ = 0.60). Bland–Altman plots further illustrate the agreement between NULISA and Simoa immunoassay–derived Alzheimer’s disease biomarkers. Overall, the biomarkers quantified using different immunoassays displayed differences clustered around 0, signifying excellent agreement between measurements from NULISA and established Simoa immunoassays (Supplementary Fig. 1, bottom panels).

### Plasma p-tau concentrations quantified using NULISA and Simoa immunoassays are associated with amyloid-PET and tau-PET

**Figure 1:**
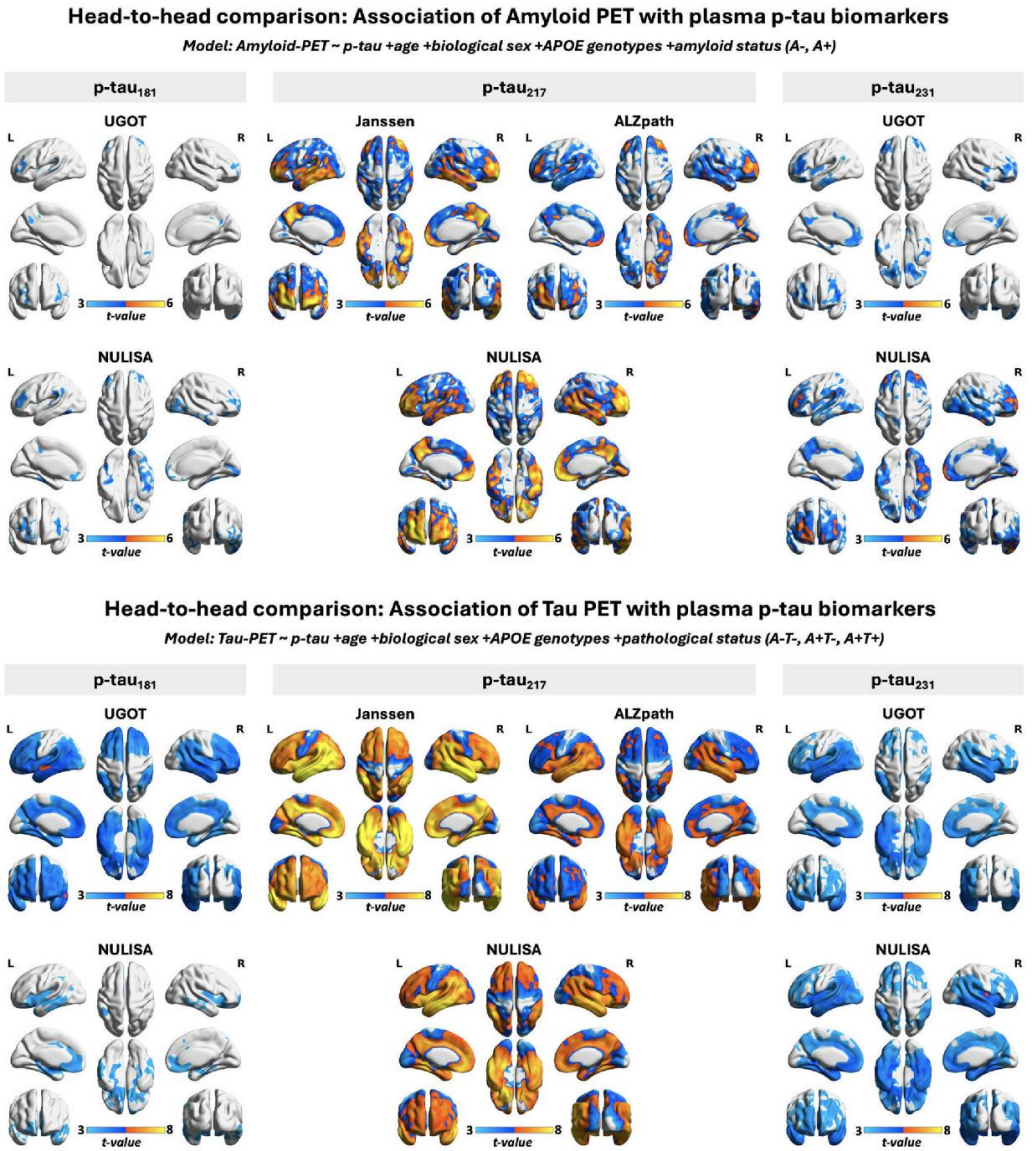
**Head-to-head comparison of the association between amyloid-PET and tau-PET with plasma p-tau concentrations measured using NULISA and Simoa immunoassays.** Voxel-based multivariate linear regression analysis (*n* = 310) revealed positive associations between amyloid-PET and tau-PET signals in the brain and the concentrations of plasma p-tau biomarkers (p-tau_181_, p-tau_217_ and p-tau_231_) quantified using different immunoassays. Age, sex and *APOEε4* carriage status were employed as covariates in the models. To account for the effects of participants’ biological stage of Alzheimer’s disease, amyloid-PET status (A− or A+) and pathological status (A−T−, A+T− or A+T+) were included as a covariate when assessing the relationship between plasma p-tau biomarkers with amyloid-PET and tau-PET, respectively. Images represent voxel-based *t*-statistical parametric maps overlaid on the structural MRI reference template. Results were also corrected for multiple comparisons using an FDR cluster threshold of *P* < 0.001.

**Figure 2:**
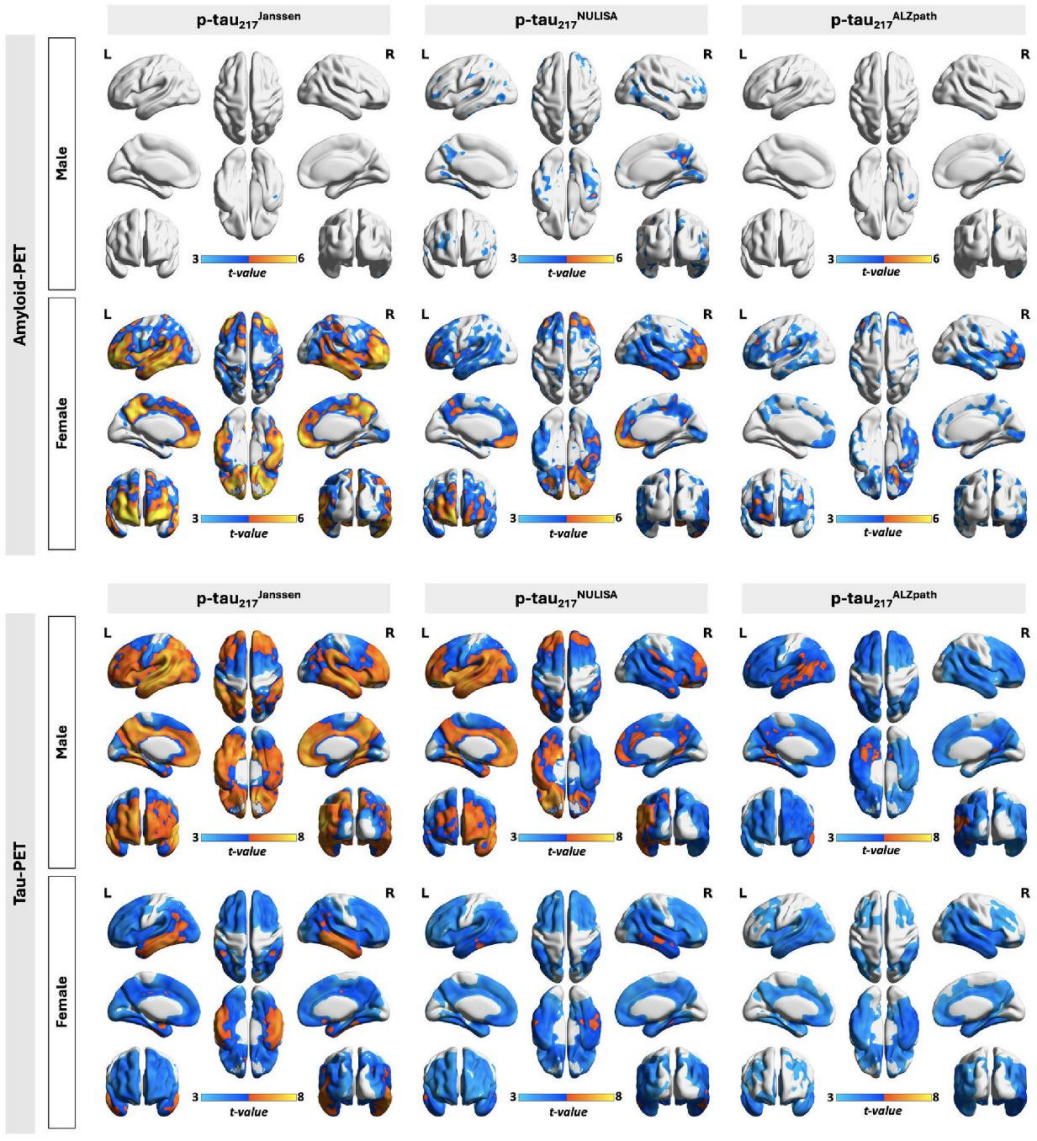
**Head-to-head comparison of the sex-specific association between amyloid-PET and tau-PET with plasma p-tau_217_ concentrations measured using NULISA and Simoa immunoassays.** Voxel-based multivariate linear regression analysis (*n* = 310) was conducted. Amyloid-PET: Positive correlations were identified between plasma p-tau_217_ concentration and amyloid-PET in female subjects, whereas this correlation disappeared in males after correcting for amyloid status. Tau-PET: Conversely, concerning the link between plasma p-tau_217_ and tau-PET, both males and females exhibit a positive association in similar brain regions. Images represent voxel-based *t*-statistical parametric maps overlaid on the structural MRI reference template. Results were corrected for age, *APOEε4* carriage status and multiple comparisons using an FDR cluster threshold of *P* < 0.001. To account for the effects of participants’ biological stage of Alzheimer’s disease, amyloid-PET status (A− or A+) and pathological status (A−T−, A+T− or A+T+) were also corrected when assessing the relationship between plasma p-tau biomarkers with amyloid-PET and tau-PET, respectively.

Voxel-based multivariate linear regression analysis demonstrated positive associations between [^18^F]AZD4694 amyloid-PET and [^18^F]MK6240 tau-PET signals in the brain and the concentrations of plasma p-tau biomarkers (Fig. 1). Compared with plasma p-tau_181_ and p-tau_231_, plasma p-tau_217_ concentrations showed a more widespread association with amyloid-PET signals in the brain. There were no significant differences observed among the three p-tau_217_ immunoassays (NULISA, Janssen and ALZpath) in this association. Similarly, plasma p-tau_217_ concentrations also exhibited a positive correlation with tau-PET signals across the brain, particularly strong in temporal and parietal areas. The sex-disaggregated analysis further provides noteworthy insights. As illustrated in Fig. 2, our findings reveal that among females, there’s a positive correlation between plasma p-tau_217_ concentrations and amyloid-PET, whereas this correlation is notably weaker in male subjects. Conversely, concerning the link between plasma p-tau_217_ and tau-PET, both males and females exhibit a positive association in similar brain regions.

### Diagnostic performance of plasma p-tau biomarkers quantified using NULISA and Simoa immunoassays

**Figure 3:**
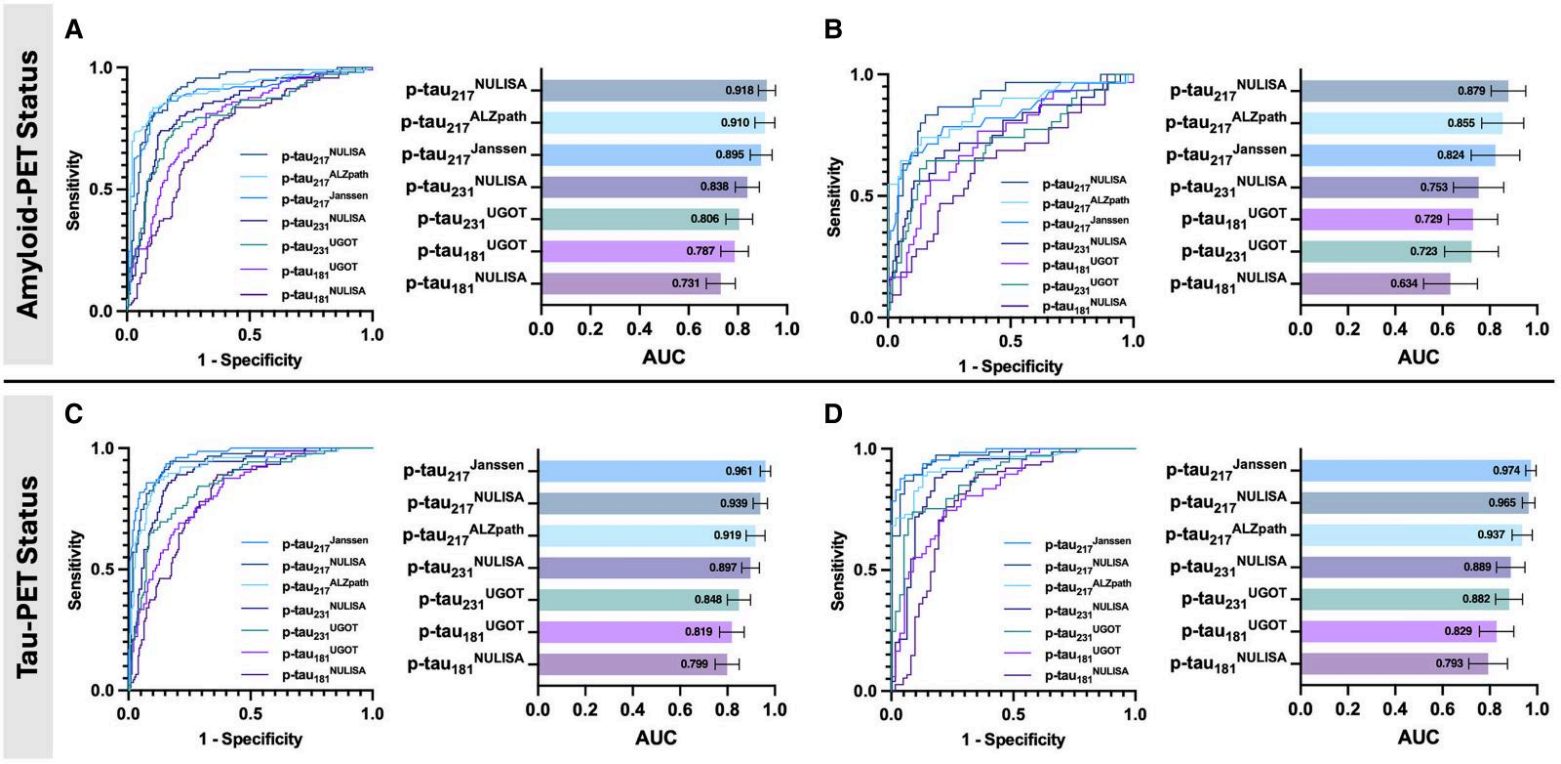
**Discriminative accuracy of NULISA and Simoa immunoassay–derived p-tau concentrations for biological Alzheimer’s disease.** ROC analyses (*n* = 310) display discriminative accuracy of plasma p-tau for amyloid-PET status and tau-PET status. (**A** and **B**) Overall, plasma p-tau_217_^NULISA^ performed the best for differentiating amyloid-PET status [(**A**) entire cohort: AUC = 0.918, 95% CI: 0.883 to 0.953, *P* < 0.0001; (**B**) CU individuals only: AUC = 0.879, 95% CI: 0.805 to 0.952, *P* < 0.0001], followed by plasma p-tau_217_^ALZpath^ (entire cohort: AUC = 0.910, 95% CI: 0.870 to 0.951, *P* < 0.0001; CU individuals only: AUC = 0.855, 95% CI: 0.765 to 0.944, *P* < 0.0001). (**C** and **D**) For discriminating tau-PET status, plasma p-tau_217_^Janssen^ showed the highest accuracy [(**C**) entire cohort: AUC = 0.961, 95% CI: 0.939 to 0.982, *P* < 0.0001; (**D**) CI individuals only: AUC = 0.974, 95% CI: 0.952 to 0.996, *P* < 0.0001) followed by plasma p-tau_217_^NULISA^ (entire cohort: AUC = 0.939; 95% CI: 0.909 to 0.969, *P* < 0.0001; CI individuals only: AUC = 0.965, 95% CI: 0.938 to 0.992, *P* < 0.0001). CI, cognitively impaired.

ROC curve analysis was performed to assess how well plasma p-tau biomarkers measured with different assays identified individuals with abnormal amyloid-PET and tau-PET status. The findings showed that plasma p-tau_217_^NULISA^ (AUC = 0.918, 95% confidence interval (CI): 0.883 to 0.953, *P* < 0.0001) and plasma p-tau_217_^Janssen^ (AUC = 0.961, 95%CI: 0.939 to 0.982, *P* < 0.0001) had the highest AUC for differentiating amyloid-PET and tau-PET status, respectively. Plasma p-tau_217_ biomarkers measured with Simoa immunoassays also showed a high differential ability for abnormal amyloid-PET and tau-PET status (Fig. 3A and C). Similar patterns of results were observed when dividing the cohort into CU and CI subgroups (Fig. 3B and D). A summary of all areas under the ROC curve values and corresponding 95% confidence intervals is presented in Table 2.

**Table 2:** Discriminative accuracy of NULISA and immunoassay-derived p-tau concentrations for biological Alzheimer’s disease

	Whole cohort		CU individuals	
	AUC (95% CI)	*P*-value	AUC (95% CI)	*P*-value
Amyloid-PET status				
p-tau_181_^NULISA^	0.731 (0.672–0.789)	<0.0001	0.634 (0.520–0.748)	0.0213
p-tau_181_^UGOT^	0.787 (0.731–0.843)	<0.0001	0.729 (0.625–0.834)	0.0001
p-tau_217_^NULISA^	0.918 (0.883–0.953)	<0.0001	0.879 (0.805–0.952)	<0.0001
p-tau_217_^Janssen^	0.895 (0.850–0.940)	<0.0001	0.824 (0.721–0.927)	< 0.0001
p-tau_217_^ALZpath^	0.910 (0.870–0.951)	<0.0001	0.855 (0.765–0.944)	< 0.0001
p-tau_231_^NULISA^	0.838 (0.790–0.887)	<0.0001	0.753 (0.647–0.859)	< 0.0001
p-tau_231_^UGOT^	0.806 (0.751–0.861)	<0.0001	0.723 (0.609–0.837)	0.0002
Tau-PET status				
p-tau_181_^NULISA^	0.799 (0.748–0.850)	<0.0001	0.793 (0.711–0.875)	< 0.0001
p-tau_181_^UGOT^	0.819 (0.767–0.870)	<0.0001	0.829 (0.756–0.901)	< 0.0001
p-tau_217_^NULISA^	0.939 (0.909–0.969)	<0.0001	0.965 (0.938–0.992)	< 0.0001
p-tau_217_^Janssen^	0.961 (0.939–0.982)	<0.0001	0.974 (0.952–0.996)	< 0.0001
p-tau_217_^ALZpath^	0.919 (0.879–0.959)	<0.0001	0.937 (0.894–0.979)	< 0.0001
p-tau_231_^NULISA^	0.897 (0.860–0.934)	<0.0001	0.889 (0.829–0.949)	< 0.0001
p-tau_231_^UGOT^	0.848 (0.800–0.897)	<0.0001	0.882 (0.825–0.939)	< 0.0001

A summary of all areas under the ROC curve values and corresponding 95% CIs for detecting abnormal amyloid-PET and tau-PET status using plasma p-tau biomarkers.

CI, Cognitively impaired.

### Predicting amyloid- and tau-PET imaging status with plasma p-tau_217_ biomarkers

**Figure 4:**
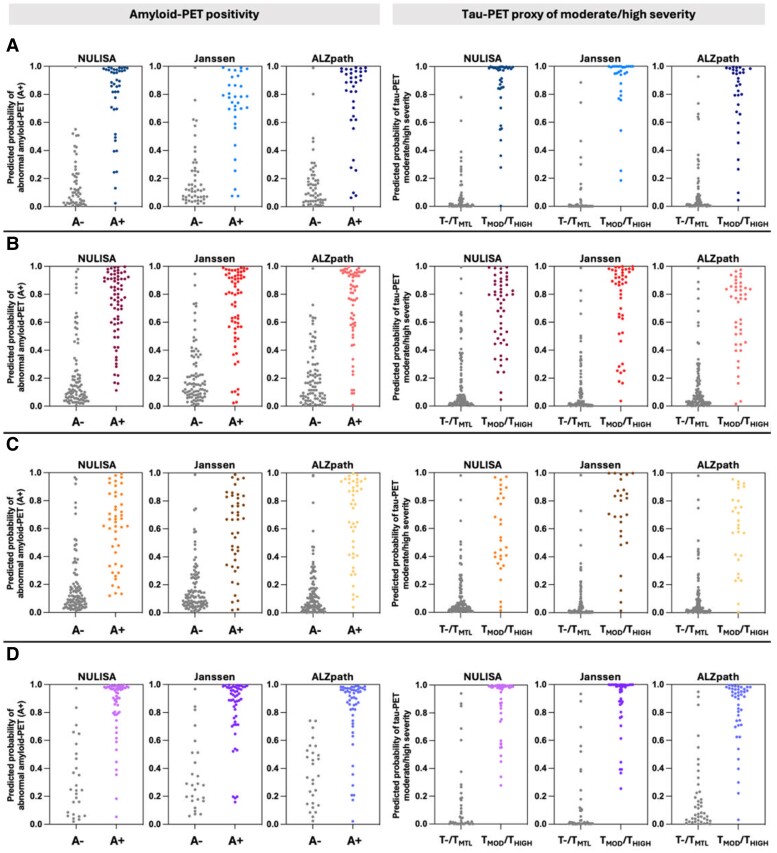
**Predicting amyloid-PET and tau-PET staging with plasma p-tau_217_ biomarkers.** The scatter plots illustrated the distribution of predicted probabilities of an abnormal amyloid-PET (left) and a tau-PET proxy of moderate/high severity (right) based on a logistic regression model (*n* = 310) including log2-transformed plasma p-tau_217_ concentrations. The predicted probabilities are displayed for (**A**) male, (**B**) female, (**C**) *APOEε4* non-carrier and (**D**) *APOEε4* carrier. The *x*-axis corresponds to individuals’ amyloid and tau status determined by PET. The *y*-axis displays the predicted probabilities of plasma p-tau_217_ biomarkers for an abnormal PET status (positivity for amyloid-PET and moderate/high severity for tau-PET). Detailed statistical information is displayed in Table 3.

Multiple logistic regression was conducted to investigate the capability of plasma p-tau_217_ biomarkers for predicting an abnormal amyloid-PET and a tau-PET proxy of moderate/high severity individually across male subjects, female subjects, *APOEε4* non-carriers and *APOEε4* carriers (Fig. 4; Table 3). Overall, plasma p-tau_217_ showed high accuracy (>85%) in classifying both amyloid-PET and tau-PET status across all groups, despite some variances observed. Specifically, as shown in Table 3, when predicting amyloid-PET abnormalities and tau-PET proxy of moderate/high severity in male subjects, plasma p-tau_217_ exhibited a specificity, positive predictive value (PPV) and negative predictive value (NPV) exceeding 85%. On the other hand, there are some trade-offs between specificity and sensitivity varied between different immunoassays in female subjects. Among *APOEε4* carriers, good specificity (>80%), sensitivity (>85%), PPV (>85%) and NPV (>75%) were observed. While in *APOEε4* non-carriers, the sensitivity was slightly compromised when predicting amyloid-PET abnormalities. Detailed statistical information from all models is illustrated in Table 3.

**Table 3:** Predicting amyloid-PET and tau-PET staging with plasma p-tau_217_ biomarkers

	Youden’s index	Specificity (%)	Sensitivity (%)	PPV (%)	NPV (%)	Accuracy (%)
Amyloid-PET abnormality						
Male						
p-tau_217_^NULISA^	0.756	98.1	77.5	96.9	85.3	89.25
p-tau_217_^Janssen^	0.717	95.9	75.8	92.6	85.5	87.80
p-tau_217_^ALZpath^	0.792	96.3	82.9	93.6	89.7	91.01
Female						
p-tau_217_^NULISA^	0.702	77.5	92.7	75.9	93.2	84.08
p-tau_217_^Janssen^	0.713	87.2	84.1	82.8	88.2	85.91
p-tau_217_^ALZpath^	0.710	91.6	79.4	87.7	85.4	86.30
APOEε4 non-carrier						
p-tau_217_^NULISA^	0.583	93.9	64.4	82.9	85.3	84.72
p-tau_217_^Janssen^	0.637	91.6	72.1	77.5	89.1	86.00
p-tau_217_^ALZpath^	0.721	92.5	79.6	81.4	91.7	88.74
APOEε4 carrier						
p-tau_217_^NULISA^	0.706	81.5	89.1	90.7	78.6	86.59
p-tau_217_^Janssen^	0.730	80.8	92.2	90.4	84.0	88.31
p-tau_217_^ALZpath^	0.722	85.7	86.5	91.8	77.4	86.25
Tau-PET proxy of moderate/high severity						
Male						
p-tau_217_^NULISA^	0.872	95.5	91.7	91.7	95.5	94.12
p-tau_217_^Janssen^	0.897	96.8	92.9	92.9	96.8	95.56
p-tau_217_^ALZpath^	0.796	92.5	87.1	84.4	93.9	90.82
Female						
p-tau_217_^NULISA^	0.707	88.7	82.0	77.4	91.3	86.54
p-tau_217_^Janssen^	0.759	96.3	79.6	89.7	92.0	91.45
p-tau_217_^ALZpath^	0.774	91.7	85.7	80.0	94.3	90.00
APOEε4 non-carrier						
p-tau_217_^NULISA^	0.769	90.7	86.2	69.4	96.4	89.80
p-tau_217_^Janssen^	0.815	96.9	84.6	84.6	96.9	94.77
p-tau_217_^ALZpath^	0.644	97.7	66.7	85.7	93.4	92.36
APOEε4 carrier						
p-tau_217_^NULISA^	0.833	87.5	95.8	90.2	94.6	92.05
p-tau_217_^Janssen^	0.829	82.9	100	86.3	100	91.76
p-tau_217_^ALZpath^	0.768	88.4	88.4	88.4	88.4	88.37

Multiple logistic regression was performed to investigate the capability of plasma p-tau_217_ biomarkers for predicting amyloid-PET abnormality and tau-PET proxy of moderate/high severity. The specificity, sensitivity, PPV and NPV were calculated based on the biomarker's maximum potential effectiveness, as indexed by Youden’s index. Youden’s index = specificity + sensitivity − 1.

## Discussion

This presents the first paper to validate the utility and performance of a novel nucleic acid–linked multiplex immunoassay technology—NULISA for protein biomarkers through head-to-head comparisons with established Simoa immunoassays in the Alzheimer’s disease field. Overall, our findings suggest an excellent agreement and a comparable discriminative accuracy for biological Alzheimer’s disease between NULISA- and Simoa immunoassay–derived plasma p-tau measurements. This novel proteomic immunoassay will add to the current analytical methods for leveraging blood-based biomarkers in Alzheimer’s disease diagnosis, screening and staging.

Blood-based biomarkers have been revolutionizing the detection, diagnosis and screening of Alzheimer’s disease.^19-22,25,26^ Compared with neuroimaging and CSF, blood-based biomarkers offer a non-radioactive, non-invasive, easily accessible and cost-effective approach and show great potential for capturing the disease’s dynamic physiological and pathological processes. In this study, we showed that p-tau variants quantified using NULISA present high concordance with other established methods (Supplementary Fig. 1) and display positive associations with amyloid-PET and tau-PET signals in the brain (Fig. 1). To our knowledge, this represents one of the first studies conducting head-to-head comparisons of the voxel-based relationship between PET signals in the brain and plasma p-tau biomarkers measured with different immunoassays. As the Alzheimer’s disease research community has transitioned towards a biological definition of the disease,^7^ the emergence of anti-Aβ therapies approved by the Food and Drug Administration (FDA) and upcoming clinical trials emphasize the importance of validated blood biomarkers for detecting abnormal Aβ and tau pathology. These biomarkers are pivotal in facilitating timely treatment decisions, ensuring patients receive suitable interventions or are recruited into pertinent clinical trials. Consistent with earlier studies demonstrating the efficacy of plasma p-tau_217_ in detecting Alzheimer’s disease pathology,^22,26-28^ ROC curve analysis in our study revealed that all three commercially available plasma p-tau_217_ assays (NULISA, Janssen and ALZpath) exhibited strong performance in identifying abnormal amyloid-PET and tau-PET status. Similar patterns of results were observed in the sub-analyses conducted within CU individuals for identifying A+ status and within CI individuals for identifying T+ status, as this is more relevant for the purposes of differential diagnosis (Fig. 3B and D).

In this present study, we delved into a crucial aspect of implementing plasma biomarkers in real-world clinical settings: the impact of Alzheimer’s disease risk factors including *APOEε4* allele(s) and female sex. *APOE* has been considered the most important genetic risk factor for sporadic Alzheimer’s disease. Researchers estimate that more than 50% of people diagnosed with Alzheimer’s disease dementia carry at least one copy of the *APOEε4* allele.^29,30^ On the other hand, females are disproportionately affected by Alzheimer’s disease, comprising approximately two-thirds of all Alzheimer’s disease patients. Given the emergence of plasma biomarkers, it is crucial to conduct investigations and report any potential sex and genetic disparities in biomarker performance. Findings from our study provide some valuable insights. Results from voxel-based analyses display positive associations between concentrations of plasma p-tau_217_ and both amyloid-PET and tau-PET signals in female participants (Fig. 2). This is in line with some previous findings indicating sex differences in the association between plasma p-tau biomarkers and Aβ,^31^ and there is a sex-specific modulation in the relationship between Aβ and tau phosphorylation.^32^ Our findings also indicated that in male participants, the relationship between plasma p-tau_217_ and amyloid-PET is highly dependent on their amyloid status, whereas this was not the case for female subjects. This difference might explain why the performance of plasma p-tau_217_ in differentiating amyloid status is better in males. Future research is needed to elucidate the contributors to the variances in the plasma p-tau_217_ concentrations in females, regardless of the amyloid status. These collectively suggest that biological sex may impact the relationships between plasma p-tau_217_ concentrations, Aβ and neurofibrillary tangle (NFT) burden in the brain, which raises caution in the sex differences when using plasma p-tau_217_ as an accessible Alzheimer’s disease biomarker and screening tool for preventive and therapeutic clinical trials.

Furthermore, we also assessed the predictive power of plasma p-tau_217_ biomarkers for an abnormal amyloid-PET and a tau-PET proxy of intermediate/high (T_MOD_/T_HIGH_) severity individually across male subjects, female subjects, *APOEε4* non-carriers and *APOEε4* carriers. This was inspired by the imaging–autopsy correlation studies, which have shown that individuals meeting the PET imaging criteria of >A^LOW^ and >Braak Stage I–II would satisfy neuropathological criteria for moderate to severe Alzheimer’s disease neuropathological change (Thal Phase II or greater and Braak Stage III or greater).^33-35^ Our findings indicated that while plasma p-tau_217_ generally exhibited high specificity, sensitivity, PPV and NPV when employed to predict a tau-PET proxy of moderate/high severity, some variations were noted in the performance of immunoassays across different groups when used to predict amyloid-PET abnormality. This highlights the need for caution when relying on a sole classification threshold for screening Aβ positivity using plasma p-tau_217_. Perhaps selecting two thresholds (lower probability threshold with high sensitivity to avoid missing detection of Aβ positive patients and higher probability thresholds with high specificity to avoid classifying Aβ negative patients as Aβ positive), which have been proposed previously, would be a strategic approach.^36^ Furthermore, future research should explore whether different thresholds are necessary to be implemented when using plasma p-tau to distinguish between amyloid and tau status in males and females, as well as between *APOEε4* non-carriers and carriers. Nevertheless, the potential of plasma p-tau_217_ to identify individuals who would likely qualify for a neuropathological diagnosis of Alzheimer’s disease using plasma biomarkers marks an important milestone and could have a significant impact in clinical settings.

Another exciting potential about NULISA is its capability of quantifying 120 analytes in a single run thereby reducing the time required for analysis and minimizing the amount of blood samples needed. This multiplexed quantification also presents an important advantage of biofluid assessments over neuroimaging, which are typically tailored to a singular target. For example, a recent study has linked the plasma levels of glial fibrillary acidic protein (GFAP), neurofilament light (NfL), growth differentiation factor 15 and latent transforming growth factor beta binding protein 2 with dementia.^37^ By integrating multiple blood-based biomarkers, the accuracy of identifying individuals at high risk for dementia can be improved, thereby facilitating early intervention efforts.

This study is conducted in a well-characterized Alzheimer’s disease research cohort with high-affinity PET imaging agents for Aβ plaques and tau neurofibrillary tangles, yet several limitations need to be noted. The first is that the demographic makeup of the TRIAD cohort is not representative of the populations at risk for dementia in North America or globally. Hence, it is imperative to replicate these findings in larger, more diverse cohorts that better represent the general population, as well as in real-world clinical settings.^38^ A second limitation is that in this current study, the immunoassays we examined are limited to NULISA and Simoa; future investigations are needed to compare other platforms such as MSD and Lumipulse.^39^ Thirdly, the NULlSAseq multiplex assay employs relative quantification, which is expressed in units of fold change. The choice between relative and absolute quantification (in absolute concentration units, such as pg/mL) depends on the goals of the studies. Relative quantification allows the comparison of protein levels between different samples or conditions. Many well-established data analysis methods exist for characterizing the expression of analytes quantified on a relative scale, and these are particularly well suited for high-plex analysis. However, although in many cases relative quantification is sufficient for protein assays, there are situations such as in clinical diagnostics, pharmacokinetics studies or when comparing protein levels across different platforms, where absolute quantification is required. Despite this cannot be achieved with the NULISA multiplex assays, the NULlSA singleplex assays, using conventional standard curve methodology, do provide precise measurement of protein concentration in absolute terms and might present as an alternative when the actual protein concentration is crucial for the study objectives. Finally, there are several practical concerns related to the feasibility of implementing NULISA or other immunoassays in clinical routines.^12,40^ Future studies should carefully evaluate the advantages and disadvantages of different assays, compare the costs of instruments and reagents, assess the assay throughput to meet demand and ensure that analytical run times align with the requirements of the clinical setting before real-world implementation.

In summary, our study demonstrates that plasma p-tau measurements derived from NULISA exhibit high concordance with those obtained from established Simoa immunoassays. Specifically, plasma p-tau_217_ shows robust positive associations with amyloid-PET and tau-PET signals in the brain, along with excellent discriminative accuracy for abnormal amyloid-PET and tau-PET status. The capacity of plasma p-tau_217_ to identify individuals likely to meet neuropathological criteria for Alzheimer’s disease diagnosis also marks a significant advance with potential implications for clinical practice. Incorporating the NULISA technology alongside existing analytical methods will facilitate further exploration into the mechanisms linking blood biomarkers and pathological changes in Alzheimer’s disease. This holds great promise for enhancing our understanding of the disease and improving diagnostic accuracy and management strategies in clinical settings.

## Supplementary Material

fcaf004_Supplementary_Data

## Data Availability

Data from the TRIAD cohort that support the findings of this study are available from the corresponding author upon reasonable request. All requests for raw and analyzed data and materials will be promptly reviewed by McGill University to verify if the request is subject to any intellectual property or confidentiality obligations. Anonymized data will be shared upon request from a qualified academic investigator for the purpose of replicating the procedures and results presented in this article. Any data and materials that can be shared will be released via a material transfer agreement. Data are not publicly available due to information that could compromise the privacy of research participants.
