# Supplementary material for: Identify biological Alzheimer’s disease using a novel nucleic acid–linked protein immunoassay
Source: Brain Commun. 2025 Jan 7;7(1):fcaf004. doi: 10.1093/braincomms/fcaf004 (PMC11753389; doi:10.1093/braincomms/fcaf004)
Supplement: fcaf004_Supplementary_Data [file fcaf004_supplementary_data.docx]

**Supplementary material**

**Supplementary Method 1. PET acquisition and processing**

Study participants underwent three-dimensional MRI scans (Siemens), along with [^18^F]AZD4694 amyloid-PET and [^18^F]MK6240 tau-PET imaging performed on the same brain-dedicated scanner (Siemens high-resolution research tomograph, HRRT). [^18^F]AZD4694 images were acquired at 40–70 min after the intravenous bolus injection of the tracer and reconstructed with an ordered subset expectation maximization (OSEM) algorithm on a four-dimensional (4D) volume with 3 frames (3 × 600 s). [^18^F]MK-6240 images were acquired at 90–110 min after the intravenous bolus injection of the tracer and reconstructed using the same OSEM algorithm on a 4D volume with 4 frames (4 × 300 s), as previously described[^28^](https://sciwheel.com/work/citation?ids=6048779&pre=&suf=&sa=0&dbf=0). At the end of each PET acquisition, a 6-min transmission scan with a rotating ^137^Cs point source was performed for attenuation correction. PET images were also corrected for motion, dead time, decay and scattered and random coincidences. Briefly, PET images were linearly registered to the native T1-weighted image space and the T1-weighted images were linearly and nonlinearly registered to the ADNI standardized space. Then, PET images in the T1 space were brought to the ADNI standard space using transformations from native MRI to the ADNI standardized space. PET images were subsequently spatially smoothed to an 8-mm full-width at half maximum resolution. [^18^F]AZD4694 standardized uptake value ratio (SUVR) used the whole cerebellum gray matter as the reference region whereas [^18^F]MK6240 SUVR used the inferior cerebellar gray matter.

**Supplementary Method 2. P-tau immunoassays methods**

**Plasma p-tau_181_^UGOT^**: The AT270 mouse monoclonal antibody (MN1050; Invitrogen, Waltham, MA, USA) specific for the threonine-181 phosphorylation site was coupled to paramagnetic beads (103207; Quanterix) and used for capture. This antibody recognizes the tau sequence 176-PPAPKT(p) P-182 phosphorylated specifically at threonine-181. As the detector, the anti-tau mouse monoclonal antibody Tau12 (806502; BioLegend, San Diego, CA, USA), which binds the N-terminal epitope 6-QEFEVMEDHAGT-18 on human tau protein was used. The amino acid numbering follows that of the full-length tau 1-441 (Uniprot ID P10636-8). The detection antibody was conjugated to biotin (A3959; Thermo Fisher Scientific, Waltham, MA, USA) following the manufacturer’s recommendations. Full-length recombinant tau1-441 phosphorylated *in vitro* by glycogen synthase kinase 3β (TO8–50FN; SignalChem, Vancouver, BC, Canada) was used as the calibrator.^1,2^

**Plasma p-tau_217_^Janssen^**: PT3 mAb (JRD/PT/3; epitope = aa210-220 of human tau, requiring phosphorylation at aa217 and enhanced binding from additional phosphorylation at aa212), HT43 mAb (JRD/hTau/43; epitope = aa7–20 of human tau), and PT82 (JRD/PT/82; epitope = aa119–126 of human tau) were obtained from Janssen. PT3 mAb was conjugated via NHS chemistry to magnetic beads (Thermo Fisher, cat#88827), then washed 2x with TBS/Tween20, then 1x with PBS/BSA (1%). Beads (50ul) were incubated with 0.5 mL serum for 1.5 hours, then washed 2x with TBS/Tween20 and 1x with water before elution with 75 μl glycine. Eluate was neutralized with 1 M Tris-Base (12% by volume) and then heated at 85◦C for 7 minutes to denature any eluted PT3 mAb. After chilling, the samples were measured at 1:4 dilution in p217+tau assay. All Simoa homebrew reagents and HD-1 instruments were obtained from Quanterix. The Janssen pTau217 assay has a limit of detection of 0.013 pg/mL.^3^

**Plasma p-tau_217_^ALZpath^**: The ALZpath pTau217 assay uses a proprietary monoclonal p-tau217 specific capture antibody, an N-terminal detector antibody, and a peptide calibrator. It has a limit of detection of 0.0052 to 0.0074 pg/mL, a functional lower limit of quantification of 0.06 pg/mL, and a dynamic range of 0.007 to 30 pg/mL.^4^

**Plasma p-tau_231_^UGOT^**: Monoclonal mouse antibodies were generated using a synthetic peptide (K224KVAVVR(pT)PPKSPSSAK240C) as a KLH-coupled antigen, numbered according to full-length tau-441 phosphorylated on threonine 231. Candidate hybridomas were selected on brain extracts of AD and control brain tissue. The final cloned and purified monoclonal antibody, ADx253, was characterized on synthetic peptides spanning amino acids threonine 217 till serine 241 of full-length tau for its affinity, its phospho-specificity using both phosphorylated and non-phosphorylated peptides and its preferred selectivity in which position 232 was replaced by a Pip, to simulate cis-selectivity of ADx253. A biotin-conjugated N-terminal anti-tau mouse monoclonal antibody was used for detection. Full-length recombinant tau 441 phosphorylated *in vitro* by glycogen synthase kinase 3β was used as the calibrator.^5^

**Supplementary Method 3. NULISAseq assay, data processing and normalization**

Plasma samples stored at -80^o^C were thawed on ice and centrifuged at 10,000g for 10 mins. 10uL supernatant samples were plated in 96-well plates and analyzed with Alamar’s CNS Disease Panel targeting mostly neurodegenerative disease-related targets as well as inflammation and immune response-related cytokines and chemokines. A Hamilton-based automation instrument was used to perform the NULISAseq workflow, starting with immunocomplex formation with DNA-barcoded capture and detection antibodies, followed by capturing and washing the immunocomplexes on paramagnetic oligo-dT beads, then releasing the immunocomplexes into a low-salt buffer, which were captured and washed on streptavidin beads. Finally, the proximal ends of the DNA strands on each immunocomplex were ligated to generate a DNA reporter molecule containing both target-specific and sample-specific barcodes. DNA reporter molecules were pooled and amplified by PCR, purified and sequenced on Illumina NextSeq 2000. For NULISAseq,^6^ sequencing data were processed using the NULISAseq algorithm (Alamar Biosciences). The sample- (SMI) and target-specific (TMI) barcodes were quantified, and up to two mismatching bases or one indel and one mismatch were allowed. Intraplate normalization was performed by dividing the target counts for each sample well by that well’s internal control counts. Interplate normalization was then performed using interplate control (IPC) normalization, wherein counts were divided by target-specific medians of the three IPC wells on that plate. Data were then rescaled, add 1 and log2 transformed to obtain NULISA Protein Quantification (NPQ) units for downstream statistical analysis. The LOD of p-tau_217_ measured with NULISA single-plex assay was reported to be 0.007 pg/mL (https://alamarbio.com/wp-content/uploads/2023/11/Alamar Poster_BUCK_1223_web.pdf).

**Supplementary Method 4. Tau-PET proxy of low and moderate/high severity**

Tau-PET SUVR in each Braak ROI was categorized as either normal or abnormal based on the mean plus 2.5 SD SUVR in that specific Braak region from CU young subjects. Participants were classified into two groups: T-/T_MTL_ (79.4%), representing subjects with tau-PET normalcy across all Braak ROIs or abnormality limited to Braak regions I-II, and T_MOD_/T_HIGH_ (20.6%), representing individuals with tau-PET abnormalities in Braak regions I-III(IV) or Braak regions I-V(VI).

**Supplementary Method References**

1. Karikari TK, Pascoal TA, Ashton NJ, et al. Blood phosphorylated tau 181 as a biomarker for Alzheimer’s disease: a diagnostic performance and prediction modelling study using data from four prospective cohorts. *Lancet Neurol*. 2020;19(5):422-433. doi:10.1016/S1474-4422(20)30071-5

2. Karikari TK, Benedet AL, Ashton NJ, et al. Diagnostic performance and prediction of clinical progression of plasma phospho-tau181 in the Alzheimer’s Disease Neuroimaging Initiative. *Mol Psychiatry*. 2021;26(2):429-442. doi:10.1038/s41380-020-00923-z

3. Triana-Baltzer G, Moughadam S, Slemmon R, et al. Development and validation of a high-sensitivity assay for measuring p217+tau in plasma. *Alzheimers Dement Diagn Assess Dis Monit*. 2021;13(1):e12204. doi:10.1002/dad2.12204

4. Ashton NJ, Brum WS, Di Molfetta G, et al. Diagnostic accuracy of a plasma phosphorylated tau 217 immunoassay for alzheimer disease pathology. *JAMA Neurol*. 2024;81(3):255-263. doi:10.1001/jamaneurol.2023.5319

5. Ashton NJ, Pascoal TA, Karikari TK, et al. Plasma p-tau231: a new biomarker for incipient Alzheimer’s disease pathology. *Acta Neuropathol (Berl)*. Published online February 14, 2021. doi:10.1007/s00401-021-02275-6

6. Feng W, Beer JC, Hao Q, et al. NULISA: a proteomic liquid biopsy platform with attomolar sensitivity and high multiplexing. *Nat Commun*. 2023;14(1):7238. doi:10.1038/s41467-023-42834-x

**Supplementary Figure 1. Relationships between NULISA and other immunoassay-measured AD biomarkers**

**Top panels**: Simple regression analysis was performed to assess the relationship between AD biomarkers quantified using NULISA and other established immunoassays. Significant positive linear relationships were observed (*P* < 0.0001; p-tau_181_^NULISA vs UGOT^: R^2^ = 0.48; p-tau_217_^NULISA vs Janssen^: R^2^ = 0.73; p-tau_217_^NULISA vs ALZpath^: R^2^ = 0.71; p-tau_231_^NULISA vs UGOT^: R^2^ = 0.60). The regression line and corresponding 95% confidence intervals are illustrated in the scatterplots. X and y axis present the log_2_-transformed biomarker concentrations (pg/mL).

**Bottom panels**: Bland–Altman analysis was conducted to assess the bias between NULISA-quantified and other immunoassay-quantified AD biomarkers. Although p-tau_181_ and p-tau_231_ exhibited a slightly larger standard deviation of bias, p-tau biomarkers overall displayed excellent agreement between measurements from NULISA and other established immunoassays (p-tau_181_^NULISA vs UGOT^: bias = 0.017 [0.78], p-tau_231_^NULISA vs UGOT^: bias = -0.021 [0.659]; p-tau_217_^NULISA vs Janssen^: bias = -0.045 [0.54]; p-tau_217_^NULISA vs ALZpath^: bias = -0.069 [0.55]). Z-scores for each biomarker (log_2_-transformed) are presented to facilitate comparisons between measurements. Dashed lines indicate 95% limits of agreement.

| **p-tau_181_ ^NULISA vs UGOT^** | **p-tau_217_ ^NULISA vs Janssen^** | **p-tau_217_ ^NULISA vs ALZpath^** | **p-tau_231_ ^NULISA vs UGOT^** |
| --- | --- | --- | --- |
| **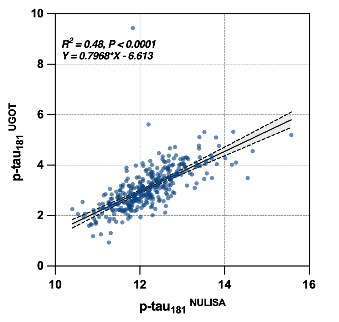** | **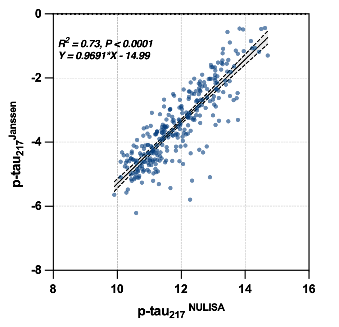** | **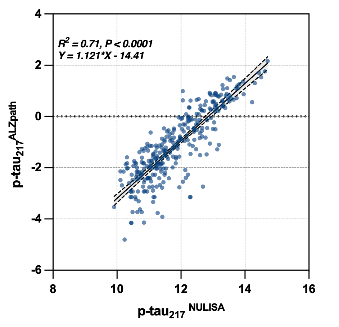** | **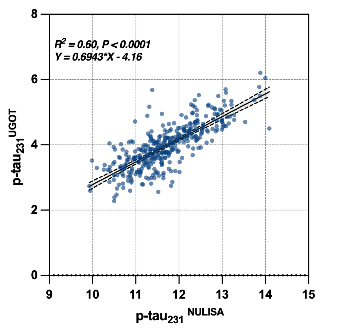** |
| **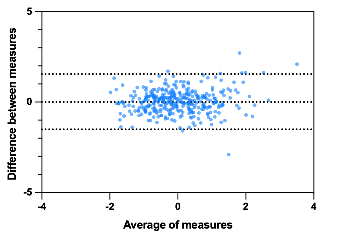** | **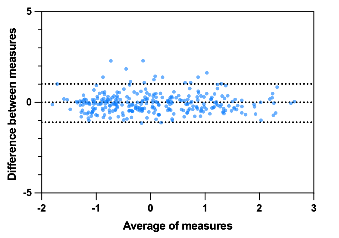** | **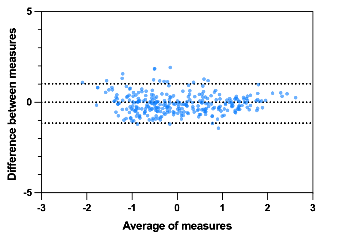** | **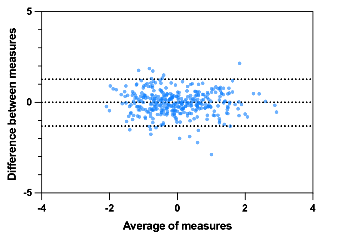** |
